# Longitudinal assessment of salivary vitamin D binding protein during orthodontic tooth movement

**DOI:** 10.1186/s12903-021-01689-8

**Published:** 2021-07-05

**Authors:** Nada Tashkandi, Yihong Zhao, Gabrielle Mitchell-Lee, Danielle Stephens, Michele Patel, Melih Motro, Leslie A. Will, Alpdogan Kantarci

**Affiliations:** 1 Department of Applied Oral Sciences, Forsyth Institute, 245 First St, Cambridge, MA 02142 USA; 2 Department of Orthodontics and Dentofacial Orthopedics, Boston University Henry M Goldman School of Dental Medicine, Boston, MA USA; 3 Department of Applied Psychology, Center of Alcohol and Substance Use Studies, School of Applied and Professional Psychology, Rutgers University, New Brunswick, Piscataway, NJ USA; 4 School of Dental Medicine, Harvard University, Boston, MA USA

**Keywords:** Vitamin D, Orthodontic tooth movement, Irregularity Index, Vitamin D Binding Protein

## Abstract

**Background:**

Vitamin D is critical for bone physiology. In this study, we quantified Vitamin D Binding Protein (VitDBP) levels in saliva as a measure of Vitamin D during orthodontic tooth movement.

**Methods:**

In this longitudinal study, saliva samples were collected from 73 orthodontic patients for 4 timepoints for the first six months of orthodontic treatment, along with dental casts at the beginning and the end of the study period. The saliva was measured for VitDBP as a biological marker for bone apposition and clinical tooth movement. We used the absolute change in Little's Irregularity Index as a quantitative measure for alignment. In addition, we measured the levels of alkaline phosphatase (ALP) in saliva as a marker of bone turnover.

**Results:**

Both low (< 2.75 ng/ml) and high (> 6.48 ng/ml) VitDBP levels were associated with reduced tooth movement. Significant (*p* < 0.05) seasonal changes in VitDBP using a two-season year model were found with lower levels observed in the summer (Apr–Sept) than in the winter (Oct–Mar).

**Conclusions:**

Clinically significant orthodontic tooth movement is associated with an optimal range of VitDBP in saliva. Normal levels of VitDBP correlated with more orthodontic tooth movement, suggesting a "normal" range of salivary content of VitDBP. Given the strong trend that is independent of the confounding factors (ex. age, race or gender), the predictive value or salivary VitDBP for tooth movement should be studied in larger cohorts in future studies.

## Background

Vitamin D (VitD) plays a significant role in bone biology and remodeling [1]. It regulates calcium absorption and the balance between osteoblasts and osteoclasts [2, 3] by producing type I collagen, alkaline phosphatase (ALP), and osteocalcin [2, 4, 5]. There are two primary forms of VitD: 25-OHD (25-hydroxyvitamin D) and 1,25-OHD [6]. After being absorbed as a pre-vitamin, VitD is transported to the liver to become hydroxylated into 25-OHD form, which is considered the dominant circulating form of the vitamin with a half-life of 2–3 weeks [7–10]. The body transforms the 25-OHD into 1,25-OHD with two hydroxylation sites [11, 12]. 1,25-OHD is considered the active hormonal form with a half-life of 4–6 h; yet, 1,25-OHD does not represent a good indicator of circulating VitD because of its short half-life and levels a thousand-fold less than 25-OHD [3, 9, 10, 13, 14].

VitD Binding Protein (VitDBP), a polymorphic single-chain serum glycoprotein ubiquitously found in body fluids and organs, has a vital role in VitD metabolism [15], binds to, solubilizes, and transports the VitD and its metabolites. Serum VitDBP concentrations and 1,25-OHD levels are positively correlated [15]. The large majority (85–90%) of circulating VitD is bound to VitDBP, 10–15% is loosely bound to serum albumin, and less than 0.03% is found in an unbound and free form [16]. VitDBP has a higher affinity with the 25-OHD form and less with the 1,25-OHD (10–100-fold lower) due to the additional hydroxyl group. VitDBP levels are estimated to be around 20-fold higher than all other VitD forms together [7]. VitDBP is less than 1% saturated, and its serum concentration (6.10^−6^ M) is substantially higher than the concentration of 25-OHD with more stability (4.10^−8^ M) [17]. Therefore, VitDBP presents an ideal candidate for the measurement of VitD in body fluids.

While several studies have focused on the association between orthodontic tooth movement and VitD levels, this study addresses the critical gap in knowledge that the orthodontic tooth movement has not been correlated with salivary levels of vitamin D binding protein as a measure of circulating vitamin D**.** [2, 3, 5, 13, 14, 18, 19]. Since saliva presents a non-invasive and practical biological milieu for biomarker detection, we hypothesized that the VitDBP levels in saliva would correlate with orthodontic tooth movement and bone turnover and that we can measure the predictive value of VitDBP during orthodontic treatment.

## Methods

### Study population

A total of 127 patients were recruited between April 2017 and March 2018 from the Department of Orthodontics and Dentofacial Orthopedics clinics at the Boston University's Henry M. Goldman School of Dental Medicine after the IRB approval (# H-34695). Seventy-three subjects were included in the final analysis. All subjects were provided with written and oral consent before being included in the study. Participants with any medical conditions that might affect bone metabolism or those taking any VitD supplements were excluded. The treatment plans included extraction and non-extraction cases, braces, clear aligner therapy, and others such as expanders or headgears.

### Measurement of alignment as a quantitative phase of orthodontic tooth movement

Impressions and orthodontic records were taken before treatment and 6 months after the start of the study period. Plaster casts were scanned using the Ortho Insight 3D from Motion View LLC., (Chattanooga, Tennessee) scanner and software. Incisor teeth were identified using the software. The mesial and distal contact points were located manually, and Little's Irregularity Index (II) [20] was measured between lower canine to canine for each pre- and post-treatment digital dental model and the absolute difference between them for statistical analysis.

### Saliva sampling and analyses

Saliva samples were collected each month for the first 6 months of orthodontic treatment. Of those, four time-points were chosed for analysis representing the the leveling and aligning stage of orthodontic treatment. The first sample was taken during the treatment planning appointment. The last sample was obtained at the end of the 6-month study phase with two samples approximately 2 and 4 months from the beginning of orthodontic treatment. Whole unstimulated saliva was collected in 15 ml polypropylene Falcon tubes at each morning appointment and immediately placed in a − 80 °C freezer. Once the collection was completed, samples were thawed and centrifuged at 12,000 rpm for 10 min, and the supernatants were aliquoted into 2 ml Eppendorf tubes. The samples collected between April and September were considered to represent "summer," and those between October and March represent "winter."

For the VitDBP measurement, a commercially available kit was used from EMD Millipore (Human Circulating Cancer Biomarker Magnetic Bead Panel 2; Cat. #HCCBP2MAG-58 K) (Burlington, Massachusetts), and samples were run on Luminex® 200™ (Austin, Texas). As a marker of bone-associated changes, we measured the ALP levels in saliva using a colorimetric ALP kit (Abcam; ab83369) (Cambridge, United Kingdom) according to the manufacturer's instructions. Optical density (OD) was measured at 405 nm by spectrophotometry. Enzyme activity was calculated as the OD of the reaction product multiplied by the reaction volume and normalized to the reaction time and the total protein. All samples were run using the same kit and the same batch simultaneously to reduce possible variation, with 10% of the samples repeated to ensure the reliability of measurements.

### Statistical analysis

Statistical analyses were performed using SAS 9.4 software. Before statistical modeling, data were thoroughly checked for potential data entry errors and outliers. Descriptive statistics were summarized using mean and standard deviation for continuous variables and frequency for categorical variables. Mixed linear regression models with random intercept were used to assess the relationship between VitDBP and season. A multiple linear regression model was used to assess the relationship between baseline VitDBP and absolute difference in tooth irregularity measures between baseline and end-of-treatment time-points.

## Results

### Sample characteristics

A total of 127 patients were recruited for the study; 84 patients completed the study, and 73 of them were included in the final analysis due to multiple missing samples and casts. There were 43 females and 30 males with an age range of 8–63 years and a mean age of 21.5 ± 11.1 years. The adult (≥ 18 years old; *n* = 36) to child (< 18 years old; *n* = 37) ratio in the study was 0.97. In the child group, the mean age was 13.2 years, and, in the adults, the mean age was 30.2 years. Racial ethnicity was self-reported, as African American (*n* = 27), Asian (*n* = 6), Caucasian (*n* = 17), and Hispanic (*n* = 23). The Angle classification of subjects was Class I (*n* = 48), Class II, including both divisions 1 and 2 (*n* = 18), and Class III (*n* = 7), which was consistent with the typical ratios seen in a university-based orthodontic clinic. No statistically significant differences were noted considering age, gender, race, or malocclusion.

### Association between VitDBP and tooth alignment

In the entire study population, baseline VitDBP was not statistically significantly related to tooth alignment's absolute change. The significance was at *p* = 0.0748, which suggested a strong trend that VitDBP could be a predictive biomarker of tooth movement. Therefore, we decided to perform an exploratory post-hoc analysis. Since there are no reports on the "normal" levels of VitDBP in saliva, we ranked the subjects by their baseline VitDBP quartile levels. Subjects with VitDBP in the middle 50% were considered "normal," while the lower 25% and higher 25% were considered as "low" or "high," respectively.

**Fig. 1:**
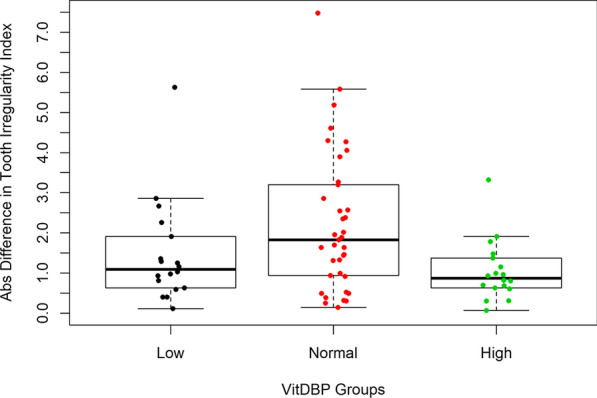
The absolute difference in tooth irregularity relationship with VitDBP groups. We found that VitDBP in the log scale was between 2.75 and 6.48 ng/ml for the "normal" range group (middle two quartiles), ≤ 2.69 ng/ml for the low subgroup (first quartile), and between 6.59 and 8.42 ng/ml for the high subgroup (fourth quartile). Our analysis demonstrated that the normal range group (mean = 2.36 ± 0.28) had a significantly higher mean absolute difference in irregularity (*p* = 0.002) than the "low" or "high" groups (mean = 1.22 ± 0.31)

Demographic information by normal vs. "low" and "high" VitDBP groups is shown in Table 1. We found that VitDBP in the log scale was between 2.75 and 6.48 ng/ml for the "normal" range group (middle two quartiles), ≤ 2.69 ng/ml for the low subgroup (first quartile), and between 6.59 and 8.42 ng/ml for the high subgroup (fourth quartile). Our analysis demonstrated that the normal range group (mean = 2.36 ± 0.28) had a significantly higher mean absolute difference in irregularity (*p* = 0.002) than the "low" or "high" groups (mean = 1.22 ± 0.31). Figure 1 shows the relationship between irregularity and VitDBP.

**Table 1:** Demographic Data of Subjects in Groups according to the LogVitDBP into "normal" range group (middle two quartiles), low subgroup (first quartile), and the high subgroup (fourth quartile)

	Low	Normal	High
Age (years)	22.78 ± 7.89	20.05 ± 11.74	23.72 ± 12.39
*Gender*			
Male	9 (12.33%)	15 (20.55%)	6 (8.22%)
Female	9 (12.33%)	22 (30.14%)	12 (16.44%)
*Race*			
African American	8 (10.96%)	12 (16.44%)	7 (9.59%)
Asian	0	4 (5.48%)	2 (2.74%)
Caucasian	2 (2.74%)	11 (15.07%)	4 (5.48%)
Hispanic	8 (10.96%)	10 (13.7%)	5 (6.85%)

### Seasonal differences in VitDBP levels

As VitD can be strongly impacted by seasonal changes due to the availability or lack of sunlight exposure, we then used a mixed regression model with a random intercept to assess the seasonal effects on VitDBP. The samples collected between April and September were considered to represent "summer," and those between October and March represent "winter." The average monthly temperature and average sunshine index were considered to distinguish between “summer” and “winter” of that year samples collected in the winter season (4.47 ± 0.30) had significantly higher VitDBP than those collected in the summer season (4.24 ± 0.30; *p* < 0.05). Overall, however, the VitDBP levels stayed in the normal range for both seasons.

### Association between VitDBP and ALP

We also examined the patterns of change in VitDBP and ALP levels across the four treatment time points. Subjects were grouped into four groups according to whether their VitDBP or ALP levels changed from quartile to quartile over various time points into changed (fluctuating), increasing, decreasing and no change. Comparing subjects with any changing patterns in VitDBP (i.e., increasing, decreasing, or fluctuating) to those in the no change group, we observed that the "no change" VitDBP group showed overall smaller mean absolute differences in irregularity index than those in the changed groups as shown in Table 2. VitDBP was significantly related to the total ALP concentration (*p* = 0.039) and the absolute change in ALP (*p* = 0.0018). Neither ALP concentration nor ALP activity was significantly related to the change in tooth movement.

**Table 2:** LogVitDBP and ALP averages over time points for all subjects. Comparing subjects with any changing patterns in VitDBP (i.e., increasing, decreasing, or fluctuating) to those in the no change group, we observed that the "no change" VitDBP group showed overall smaller mean absolute differences in irregularity index than those in the changed group

Variables	Time	Overall	Change	No change
logVitDBP (log ng/ml)	1	4.24 ± 2.12	3.91 ± 1.71	5.23 ± 2.86
	2	4.4 ± 2.13	4.13 ± 1.85	5.23 ± 2.73
	3	4.09 ± 1.89	3.75 ± 1.28	5.12 ± 2.9
	4	4.27 ± 2.1	3.97 ± 1.76	5.18 ± 2.77
ALP (ng/ml)	1	8.96 ± 9.41	7.38 ± 7.27	13.8 ± 13.21
	2	10.13 ± 9.5	10.08 ± 8.91	10.27 ± 11.39
	3	8.52 ± 7.27	7.29 ± 5.05	12.21 ± 11.05
	4	10.08 ± 7.58	9.75 ± 7.05	11.11 ± 9.18
ALP activity (µM/min/mg protein)	1	1.87 ± 1.96	1.54 ± 1.51	2.87 ± 2.75
	2	2.11 ± 1.98	2.1 ± 1.86	2.14 ± 2.37
	3	1.77 ± 1.51	1.52 ± 1.05	2.54 ± 2.3
	4	2.1 ± 1.58	2.03 ± 1.47	2.31 ± 1.91

VitDBP was significantly related to the total ALP concentration (*p* = 0.039) and the absolute change in ALP (*p* = 0.0018). Neither ALP concentration nor ALP activity was significantly related to the change in tooth movement

## Discussion

In this study, we aimed to determine whether salivary levels of VitDBP were associated with the rate of orthodontic tooth movement. We also identified the impact of confounding factors such as the seasonal effects on VitDBP levels, measured the change in VitDBP and ALP levels in response to the first six months of orthodontic treatment (leveling and aligning phase), and determined the correlation between salivary VitDBP and ALP levels during orthodontic tooth movement. The results demonstrated that "normal" levels of VitDBP in saliva were associated with a greater tooth alignment rate and resolution of crowding during orthodontic tooth movement. On the other hand, "low" and "high" levels of VitDBP were associated with a reduced orthodontic tooth movement rate. These data suggested that optimal levels of VitD may be required for orthodontic therapy.

While the role of VitD in bone metabolism is well-established [3, 4, 13, 21], there is a lack of consensus on how to measure the VitD correctly and which form of the VitD is abundant and stable in biological fluids [6, 13, 14]. The majority of the literature is focused on 25-OHD as a more stable form with a longer half-life (10–15 days) compared to 1,25-OHD (4–6 h) and at a serum concentration 1000-fold level higher than 1,25OHD [2, 7, 8, 12, 22, 23]. However, less than 0.03% of 25-OHD is unbound, which brings up the difficulty of determining the accurate levels of 25-OHD from the measurements [7, 24]. VitDBP presents a novel and reliable measure to determine the free 25-OHD_3_ [25–28]. Being a more recent marker of VitD levels, there are no international standardized levels for VitDBP in human saliva or serum currently [29–32]. Serum levels are usually estimated between 300 and 600 mg/L in healthy subjects [15]. However, there are no studies of VitDBP levels in saliva to determine the "normal" levels of VitDB. In this study, we addressed this lack of knowledge. VitDBP levels in an orthodontic population of healthy individuals without any known systemic diseases were between 0 and 8.42 ng/ml. Our results demonstrated that VitDBP could be reproducibly measured in all samples from our patient cohort at every time point, which positions VitDBP as a potential biomarker in saliva associated with oral or systemic conditions. Since we did not collect any blood specimens, the correlation of salivary VitDBP to the serum or plasma levels is not known and needs to be studied.

VitD is considered as an "accelerator" and a stabilizing agent in the orthodontic tooth movement [1, 33, 34, 36]. Local injections of 1,25-OHD in cats showed a 60% increase in tooth movement [5]. Bone regeneration was also enhanced after local and systemic VitD supplementation in dogs [25]. Accelerated tooth movement and increased number of osteoclasts and osteoblasts were associated with VitD injection in rats [2, 10, 35]. In a clinical split-mouth study, there was a decreased amount of tooth movement in humans after injection with VitD_3_ [19]. In humans, VitD may not speed up tooth movement, but physiologic levels of VitD are needed to avoid counter effects [37, 38]. As pointed out above, however, methodological issues thus far limited the use of VitD as a marker to predict orthodontic treatment outcomes. In this study, baseline values of VitDBP showed a strong trend of correlation to tooth alignment's absolute change (*p* = 0.0748). While our study demonstrated a trend, the predictive value of salivary VitDBP for tooth movement was not statistically significant. Yet, given the string trend that is independent of the confounding factors (ex: age, gender or race) this issue should be studied further in larger cohorts in future studies.

As there were no normal levels of VitDBP available for saliva, we also ranked the subjects by their VitDBP levels. Measurements were grouped according to the average VitDBP levels of the subjects rather than the time-points measured. The subjects whose levels were within the middle 50% were considered “normal” and those above or below were considered as “high” or “low”. The subjects in the “normal’ range had more significant tooth movement than those in the other groups. Normal salivary VitDBP was between 2.75 and 6.48 ng/ml, which showed a tight interval. Normal VitDBP levels in saliva significantly correlated with a higher resolution of tooth irregularity than "high' or "low" groups. This finding is striking as it demonstrates that an optimal range of VitD is necessary for predictive orthodontic treatment. Therefore, for individuals with suboptimal (low or high) VitD levels at the start of the orthodontic therapy, a customized treatment strategy can be planned, and completion of the orthodontic treatment would be predictably longer.

Another important finding in our work was that VitDBP did not demonstrate significant variation between race, age, and gender, further supporting the value of salivary VitDBP as a measure of VitD. There has been controversy in whether VitD levels vary according to race, age, and gender. African Americans and Asians were thought to have higher VitD deficiency than Caucasians with stronger overall bone health [25, 39]. Most of these studies focused on detecting 1,25-OHD or 25-OHD, which could be a potential reason for the controversy. While the studies on VitDBP are scarce, one report in line with our findings presented no racial differences in average serum concentrations of VitDBP between whites and blacks [17] supporting the notion that VitDBP is a better marker of VitD metabolism than the 1,25-OHD and 25-OHD isoforms. Likewise, associations between VitD levels and aging and gender are controversial [15]. The age range of this study was kept deliberately wide to include as many patients as possible into the study. However, statistical tests were conducted to see if there were any differences in the age groups in the tested values with no significance in any measurement which is why they were ultimately combined. Postmenopausal women had lower VitDBP levels than premenopausal women [33, 40]; VitDBP was also negatively correlated with age in females than males [26]. Women were found to have higher mean VitDBP levels than men, but no other associations were noticed between VitDBP levels and body weight, BMI, fat mass, or fat percentage [17, 40]. Other studies have shown differences in VitD and VitDBP levels with age, gender, and race [17, 25, 33, 40]. While it was not the focus in our study, there was no impact of age, gender, and race detected on salivary VitDBP levels, which requires further investigation.

Previous studies have shown that there was a significant seasonal fluctuation in 25-OHD and 1,25-OHD levels [41]. During the winter, both free and calculated serum 25-OHD levels were higher than in the summer ﻿(0.020 ± 0.005% vs. 0.019 ± 0.004%; *p* = 0.007 with no significant differences in VitDBP levels [41]. Our study found a significant (*p* < 0.05) difference in salivary VitDBP levels when considering a two-season year model. During the "winter" season defined as October to February, salivary VitDBP levels were statistically higher than in the "summer" season defined as March to September, correlating with changes noted in 25-OHD and 1,25-OHD. Further studies are needed to determine the seasonal effects on VitDBP levels and its activity on 25-OHD and 1,25-OHD levels.

Previous research has focused on the ALP as a biomarker of osteoblastic activity that could reflect tissue responses during orthodontic tooth movement [42]. There is no consensus on the normal salivary ALP levels or ALP activity [43–45]. Limited studies focused on salivary ALP levels in regards to orthodontic tooth movement [43–45, 47]. We found the mean ALP concentration levels to be 9.33 ± 8.47 pg/ml and ALP activity 1.94 ± 1.76 uM/min/mg protein. ALP concentration and activity were not significantly correlated with the absolute change in irregularity over the six months in line with a similar study where ALP levels appeared to be stable and showed no differences between time points in orthodontic patients [46, 47]. There was, however, a correlation between the VitDBP levels and ALP levels in saliva, suggesting a direct role of VitDBP in bone metabolism.

## Conclusion

Clinical outcomes of orthodontic treatment are associated with the level of VitDBP in saliva. Normal levels of VitDBP are shown to have higher orthodontic tooth movement levels than higher or lower levels. Given the strong trend that is independent of the confounding factors (ex. age, race or gender), the predictive value or salivary VitDBP for tooth movement should be studied in larger cohorts in future studies.

## Data Availability

All data and material will be available for review by contacting the corresponding author.

## Abbreviations

VitD: Vitamin D
ALP: Alkaline phosphatase
25-OHD: 25-Hydroxyvitamin D
1,25-OHD: 1,25-Hydroxyvitamin D
VitDBP: Vitamin D Binding Protein
